# Clinical characteristics and survival of second primary breast carcinoma with extramammary malignancies

**DOI:** 10.3389/fonc.2023.1160370

**Published:** 2023-03-17

**Authors:** Yaoyao Jing, Xiaofang Wang, Bei Sun

**Affiliations:** ^1^ Department of Day Ward, Tianjin Medical University Cancer Institute and Hospital, Tianjin, China; ^2^ Tianjin Medical University Cancer Institute and Hospital, National Clinical Research Center for Cancer, Tianjin, China; ^3^ Tianjin’s Clinical Research Center for Cancer, Tianjin, China; ^4^ Key Laboratory of Breast Cancer Prevention and Therapy, Tianjin Medical University, Ministry of Education, Tianjin, China; ^5^ Key Laboratory of Cancer Prevention and Therapy, Tianjin, China; ^6^ Department of Hematology, Tianjin Medical University Cancer Institute and Hospital, Tianjin, China

**Keywords:** second primary breast carcinoma, multiple primary malignant tumors, breast metastases, prognosis, survival

## Abstract

**Objectives:**

To investigate the characteristics, diagnosis, survival and prognosis of second primary breast carcinoma (SPBC).

**Materials and methods:**

Records of 123 patients with SPBC in Tianjin Medical University Cancer Institute & Hospital between December 2002 and December 2020 were retrospectively reviewed. Clinical characteristics, imaging features and survival were analyzed and comparisons between SPBC and breast metastases (BM) were made.

**Results:**

Of 67156 newly diagnosed breast cancer patients, 123 patients (0.18%) suffered previous extramammary primary malignancies. Of the 123 patients with SPBC, approximately 98.37%(121/123)were female. The median age was 55 years old (27-87). The average diameter of breast mass was 2.7 cm (0.5-10.7). Approximately 77.24% (95/123) of the patients presented with symptoms. The most common types of extramammary primary malignancies were thyroid, gynecological cancers, lung, and colorectal. Patients with the first primary malignant tumor of lung cancer were more likely to develop synchronous SPBC, and those with the first primary malignant tumor of ovarian cancer were more likely to develop metachronous SPBC. When comparing with BM, patients with SPBC were more often older (≥45 years old), at earlier stages (I/II), more microcalcification and less multiple breast masses in imaging. More than half (55.88%) of patients in the metachronous group developed primary breast cancer within 5 years after diagnosis of extramammary primary cancer. The median overall survival time was 71 months. Within 90 months, the prognosis of patients with synchronous SPBC was worse than that of patients with metachronous SPBC (*p*=0.014). Patients with BM had the worst outcome compared with patients with synchronous SPBC and metachronous SPBC (p<0.001).ER/PR-negative status, an interval of less than 6 months between the onset of two tumors, a late stage of first primary malignancy, and an age of diagnosis of first primary malignancy greater than 60 years predicted a worse prognosis for patients with SPBC.

**Conclusion:**

The possibility of SPBC should be considered during the follow-up of patients with primary extramammary malignancy, especially within 5 years of the onset of the first tumor. The stage of first primary malignancy and the age at diagnosis of first primary malignancy have an impact on the prognosis of patients with SPBC.

## Introduction

Multiple primary malignant tumors (MPMTs) are defined as malignant tumors with two or more different histological features in the same individual at the same or different times, excluding metastasis or recurrence (1). The development of MPMTs is associated with genetic predisposition, potential immune deficiencies, history of chemoradiotherapy, and exposure to carcinogens (2, 3).The development of diagnostic and improved treatments for cancer contributes to an increase in the number of cancer survivors and makes it possible for cancer patients to live long enough to develop a second primary tumor (4–7).The second primary carcinoma is an important prognostic factor among patients who survive a prior cancer, and is estimated to be the sixth most common malignant tumor worldwide (8).Cancer survivors account for 3.5% of the total population in the United States, and approximately 10% of newly diagnosed malignant tumors develop in cancer survivors (9).

Previous studies have shown that different treatments for breast malignancy could play a crucial role in the development and progression of primary extramammary cancer as delayed effects and consequences of treatment (10–12).However, only a few studies have described the types and incidence of extramammary tumors that predate the diagnosis of breast cancer. Although breast cancer has been mentioned in some studies of MPMTs, the focus is not on second primary breast cancer (3, 13, 14).In our study, we identified possible risk factors for the development of second primary breast cancer in patients with previous extramammary malignancies and assessed the survival and clinical characteristics of patients with second primary breast cancer. The differential diagnosis of primary and metastatic tumors is also important, resulting in better clinical outcomes in cases of primary cancers. We also compared SPBC and BM.

## Materials and methods

A total of 67156 breast cancer patients were newly diagnosed in Tianjin Medical University Cancer Institute & Hospital between December 2002 and December 2020.The diagnosis of all patients was confirmed by histopathology. Of these patients, 123 cases had survived a previous primary extramammary malignancy. According to the diagnosis time from the primary extramammary malignancy to the primary breast malignancy, these 123 patients were classified into the synchronous SPBC group (interval within 6 months) or the metachronous SPBC group (interval longer than 6 months) (15). Seventeen patients with pathologically confirmed BM were also included in the study, as BM group, and compared with SPBC. We retrospectively collected data on clinical characteristics, imaging features, prognosis, and pathology of previous primary malignancies and the interval time between the occurrence of two types of tumor. Family history of cancers refers to the diagnosis of cancer in a first-degree relative of patient. Studies involving human participants were approved and reviewed by the Medical Ethics Committee of Tianjin Medical University Cancer Institute & Hospital.

A total of 109 patients with SPBC and 13 with BM were followed up. We recorded and analyzed metastasis, recurrence, and survival status. Overall survival (OS) was defined as the date from the diagnosis of SPBC to the date of death from any cause or the cut-off date of follow-up (September 30, 2022).

Data comparisons for categorical variables were analyzed using two-sided Fisher’s exact test or the chi-square test. The OS of the patients was calculated using the Kaplan–Meier method. The influence of different factors on OS was analyzed using log-rank test and landmark analysis. A Cox regression model was used for multivariate survival analysis. Differences were considered statistically significant at *p* < 0.05. Statistical analyses were performed using SPSS 27.0, Graphpad prism 9.5 and R software (version 4.2.2).

## Results

### Patients’ characteristics

SPBC accounted for 0.18% (123/67156) of all breast cancers between December 2002 and December 2020.As shown in **Table 1**, the clinical characteristics of the SPBC and BM groups were compared. The median patient age was 55 (range 27-87 years of age), and 121 patients(98.37%)were female. The average diameter of breast mass was 2.7 cm (0.5-10.7). Of the patients, 76.42% were ER/PR positive and 30.08% were HER-2 positive. A family history of malignancies and smoking history were found in 42.28% and 6.50%of patients, respectively. There were 58 (47.15%) patients who had received chemoradiotherapy for their first primary malignancy. Approximately 77.24% (95/123) of the patients presented with symptoms, of which lumps(s) were the most common, accounting for 69.92% (86/95). A total of 122 patients (99.19%) had no distant metastases at the time of SPBC diagnosis. Regarding the first primary malignancies, the majority of patients (73.17%) were elderly at the time of diagnosis, and most (71.54%) were in the early stages (I/II).

**Table 1:** Clinical characteristics of patients with second primary breast carcinoma and with those of breast metastases.

		Second primary breast carcinoma (n=123), n (%)	Breast metastases (n=17), n (%)	*P* value
Gender	Male	2 (1.63%)	0 (0%)	1.000
	Female	121 (98.37%)	17 (100%)	
Age	<45	19 (15.45%)	7 (41.18%)	0.018
	≥45	104 (84.55%)	10 (58.82%)	
	Median	55	44	
	Range	29-87	27-85	
Size of breast mass (cm)	<2	36 (29.27%)	1 (5.88%)	0.094
	2-5	77 (62.60%)	14 (82.35%)	
	>5	10 (8.13%)	2 (11.76%)	
ER/PR status	Positive	94 (76.42%)	1 (5.88%)	<0.001
	Negative	29 (23.58%)	16 (94.12%)	
HER-2 status	Positive	37 (30.08%)	2 (11.76%)	0.153
	Negative	86 (69.92%)	15 (88.24%)	
Smoking history	Yes	8 (6.50%)	2 (11.76%)	0.349
	No	115 (93.50%)	15 (88.24%)	
Family history of cancers	No	71 (57.72%)	12 (70.59%)	0.431
	Yes	52 (42.28%)	5 (29.41%)	
	Breast cancer	14 (11.38%)	1 (5.88%)	
	Others	38 (30.89%)	4 (23.53%)	
History of radiotherapy or chemotherapy	Yes	58 (47.15%)	13 (76.47%)	0.023
	No	65 (52.85%)	4 (23.53%)	
Symptoms	No	28 (22.76%)	1 (5.88%)	0.197
	Yes	95 (77.24%)	16 (94.12%)	
	Lump (s)	86 (69.92%)	16 (94.12%)	
	Nipple discharge	7 (5.69%)	0 (0%)	
	Diffuse swelling	2 (1.63%)	0 (0%)	
Distant metastasis	Yes	1 (0.81%)	15 (88.24%)	<0.001
	No	122 (99.19%)	2 (11.76%)	
Interval time (months)	Synchronous	55 (44.72%)	8 (47.06%)	0.856
	Metachronous	68 (55.28%)	9 (52.94%)	
	Mean	51.6	19.8	
	Range	0-436	0-122	
Stage of first malignancy	I/II	88 (71.54%)	2 (11.76%)	<0.001
	III/IV	35 (28.46%)	15 (88.24%)	
Age at diagnosis of first malignancy	<60	33 (26.83%)	13 (76.47%)	<0.001
	≥60	90 (73.17%)	4 (23.53%)	
	Mean	53.0	48.5	
	Range	25-86	25-75	

ER: Estrogen receptor, PR: Progesterone receptor, HER-2: Human epidermal growth factor receptor 2.

### Treatment and pathology of SPBC

Of the 123 patients with SPBC, 112 underwent surgery, of which 13 received neoadjuvant chemotherapy before surgery and 58 received radiotherapy or chemotherapy after surgery. Nine patients were treated with radiotherapy or chemotherapy alone, and two refused treatment. In addition, patients with positive ER/PR and HER-2 status received endocrine therapy and targeted therapy, respectively. With regard to the treatment of the first primary malignant tumor, 101 patients received comprehensive treatment based on surgery, 18 patients received chemotherapy-based treatment, and four patients received radiotherapy alone.

The pathology of patients with SPBC included invasive ductal carcinoma (n=93, 75.61%), invasive lobular carcinoma (n=24, 19.51%), medullary carcinoma (n=2), mucinous carcinoma (n=2), mixed mucinous carcinoma (n=1), and invasive micropapillary carcinoma (n=1).

### Primary extramammary malignancy in patients with SPBC

**Figure 1:**
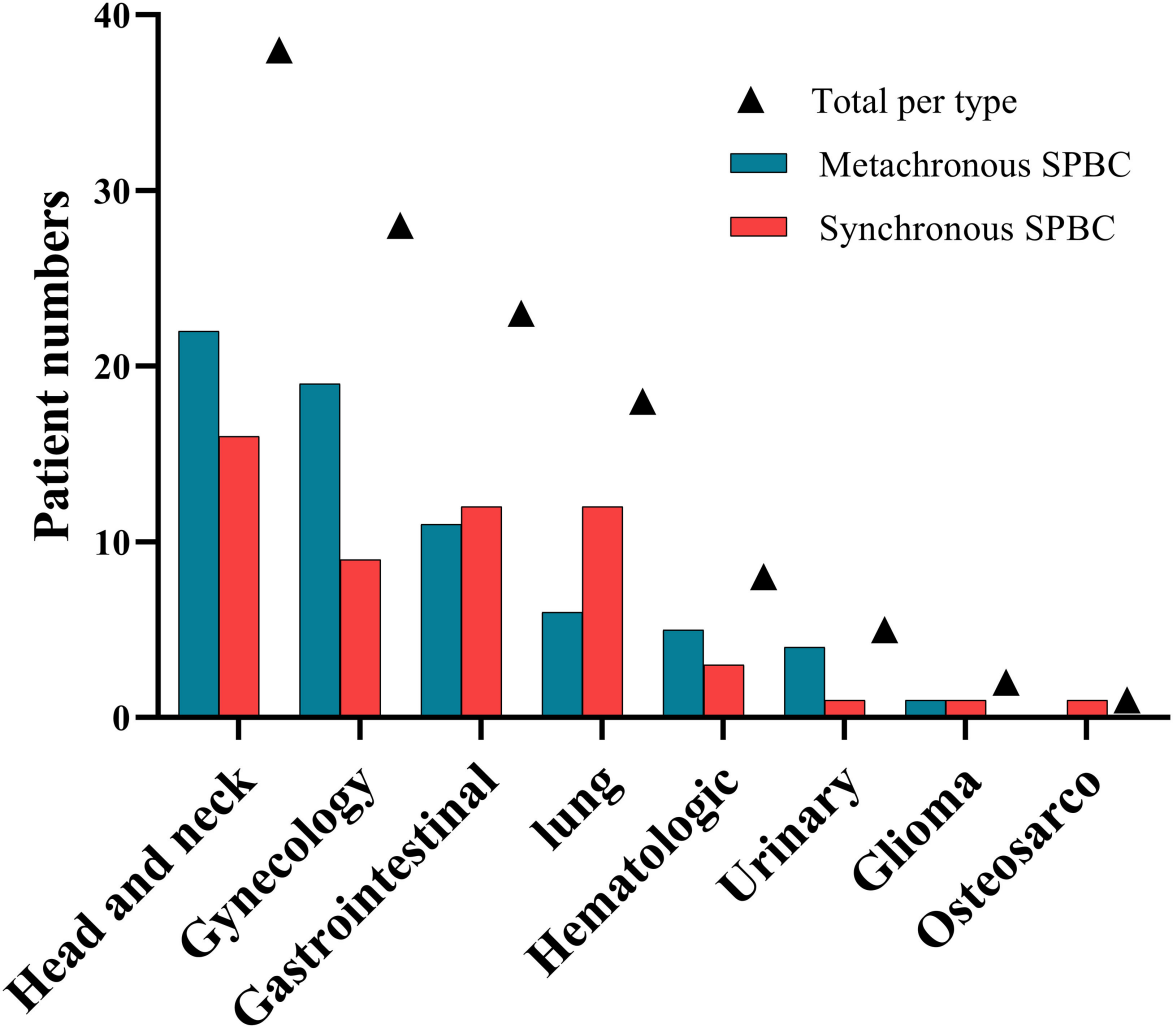
Types of primary non-breast malignancy in patients with second primary breast carcinoma.

Of the 123 patients with SPBC, 68 (55.28%) had metachronous malignancies and 55 (44.72%) had synchronous malignancies. Most of the patients had double primary malignant tumors, except eight patients who developed additional malignancies after the diagnosis of SPBC, including three triple primary cancers in the metachronous group and four triple primary cancers in the synchronous group. We observed a rare case of quadruple primary cancer in the synchronous group. The types and number of primary non-breast malignancies in patients with SPBC are shown in **Table 2** and **Figure 1**. The most common types of extramammary primary malignancies were thyroid (30.1%), gynecological (22.8%), and lung (14.6%), followed by colorectal (8.9%), hematologic (6.5%), gastric (4.1%), kidney (4.1%), and liver (3.3%) malignancies, with less common types including gallbladder (1.6%), esophageal (0.8%), oral (0.8%), glioma (1.6%), and osteosarcoma (0.8%). In the metachronous group, the most common types of first primary malignant tumors were head and neck, gynecology, and gastrointestinal, while in synchronous group, head and neck, gastrointestinal, and lung were the most common types. We further compared the distribution of the first primary malignant tumor type between the two groups and found that lung cancer was more common in the synchronous group than in the metachronous group (*p* =0.043), whereas ovarian cancer was more common in the metachronous group than in the synchronous group (*p* =0.040). This result suggests that, for women with lung cancer, targeted follow-up is needed within 6 months of diagnosis to alert them to the development of primary breast cancer. However, for patients with ovarian cancer, focused follow-up should be performed 6 months after diagnosis.

**Table 2:** Numbers and types of primary non-breast malignancy in patients with second primary breast carcinoma.

Cancer types		Synchronous (n=55), n (%)	Metachronous (n=68), n (%)	*P* value
Head and neck	Total	16 (29.09%)	22 (32.35%)	0.697
	Thyroid	16 (29.09%)	21 (30.88%)	0.829
	Oral	0 (0.00%)	1 (1.47%)	1.000
Lung		12 (21.82%)	6 (8.82%)	0.043
Gastrointestinal	Total	12 (21.82%)	11 (16.18%)	0.425
	Colorectal	4 (7.27%)	7 (10.29%)	0.753
	Gastric	3 (5.45%)	2 (2.94%)	0.656
	Esophageal	1 (1.82%)	0 (0.00%)	0.447
	Liver	3 (5.45%)	1 (1.47%)	0.324
	Gallbladder	1 (1.82%)	1 (1.47%)	1.000
Urinary	Total	1 (1.82%)	4 (5.88%)	0.379
	Kidney	1 (1.82%)	4 (5.88%)	0.379
Gynecology	Total	9 (16.36%)	19 (27.94%)	0.128
	Ovary	3 (5.45%)	12 (17.65%)	0.040
	Cervix	4 (7.27%)	4 (5.88%)	1.000
	Uterus	1 (1.82%)	3 (4.41%)	0.627
	Fallopian tube	1 (1.82%)	0 (0.00%)	0.447
Hematologic	Total	3 (5.45%)	5 (7.35%)	0.730
	Lymphoma	2 (3.64%)	2 (2.94%)	1.000
	Leukemia	0 (0.00%)	3 (4.41%)	0.252
	Polycythemia vera	1 (1.82%)	0 (0.00%)	0.447
Glioma		1 (1.82%)	1 (1.47%)	1.000
Osteosarcoma		1 (1.82%)	0 (0.00%)	0.447

### Intervals between primary extramammary malignancy and SPBC

**Figure 2:**
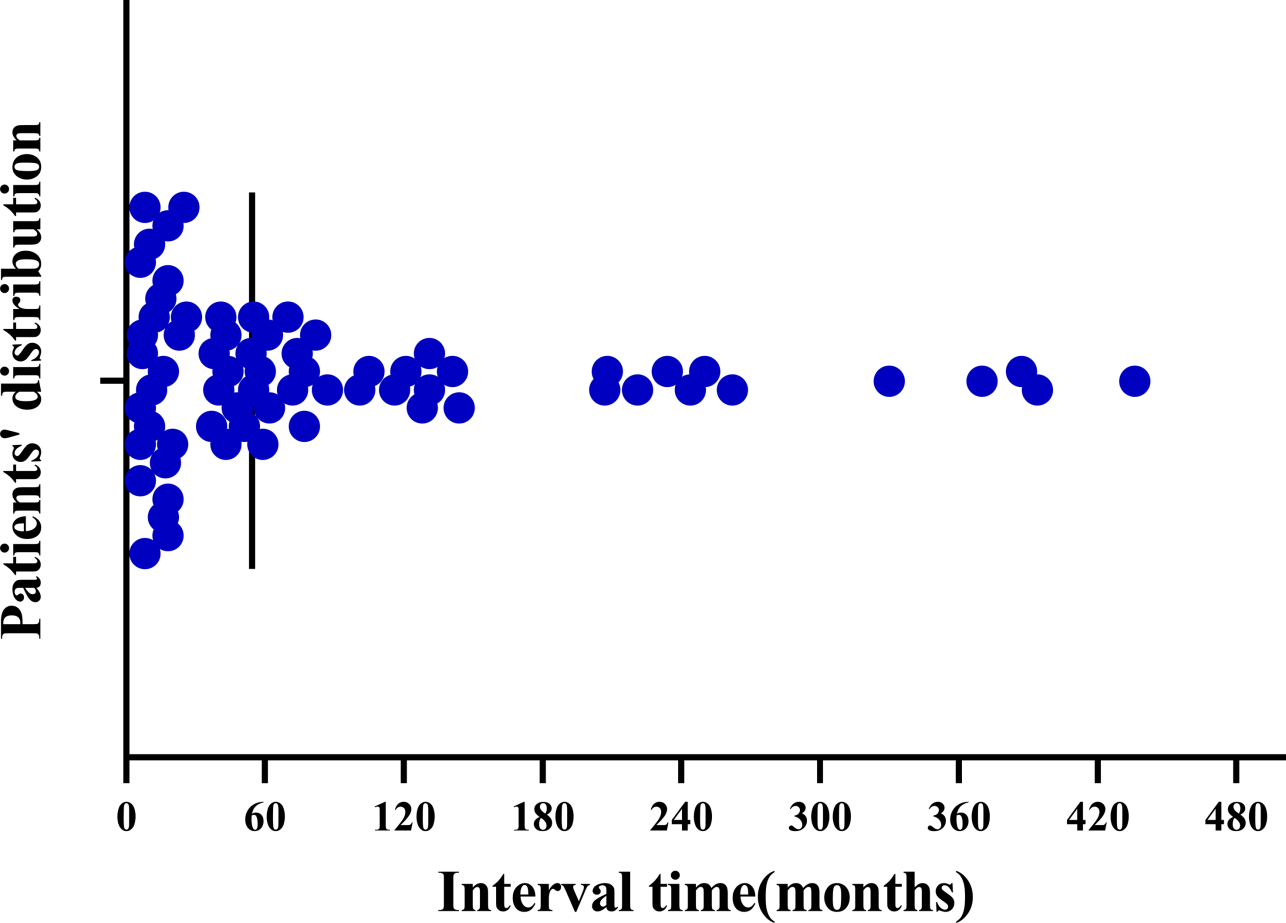
Distribution of time intervals between the development of metachronous second primary breast cancer in patients with primary non-breast malignancy.

For the 123 patients included, the mean interval between the onset of primary non-breast malignancy and SPBC was 51.6 months (range, 0-436 months). Among the 68 patients in the metachronous group, the mean duration between the onset of the two cancers was 92.9 months. As shown in **Table 3** and **Figure 2**, in the metachronous group, more than half (55.88%) of the patients developed primary breast cancer within 5 years after diagnosis of the first primary extramammary malignancy. Fortunately, the risk of developing SPBC decreased over time (**Figure 2**). The longest interval observed was nearly 37 years. This result suggests that patients should be closely followed for a second primary breast cancer within 5 years of the diagnosis of extramammary malignancy, and that the frequency of follow-up could be reduced, but not interrupted, 5 years after the diagnosis of the first extramammary malignancy.

**Table 3:** Average interval time for different cancer types to develop second primary breast carcinoma.

Metachronous interval time (years)	Number	Percentage
0-5	38	55.88%
5-10	12	17.65%
10-15	6	8.82%
15-20	4	5.88%
20-25	3	4.41%
25-30	1	1.47%
≥30	4	5.88%
Total	68	100%

### Survival and prognostic factors of patients with SPBC

**Figure 3:**
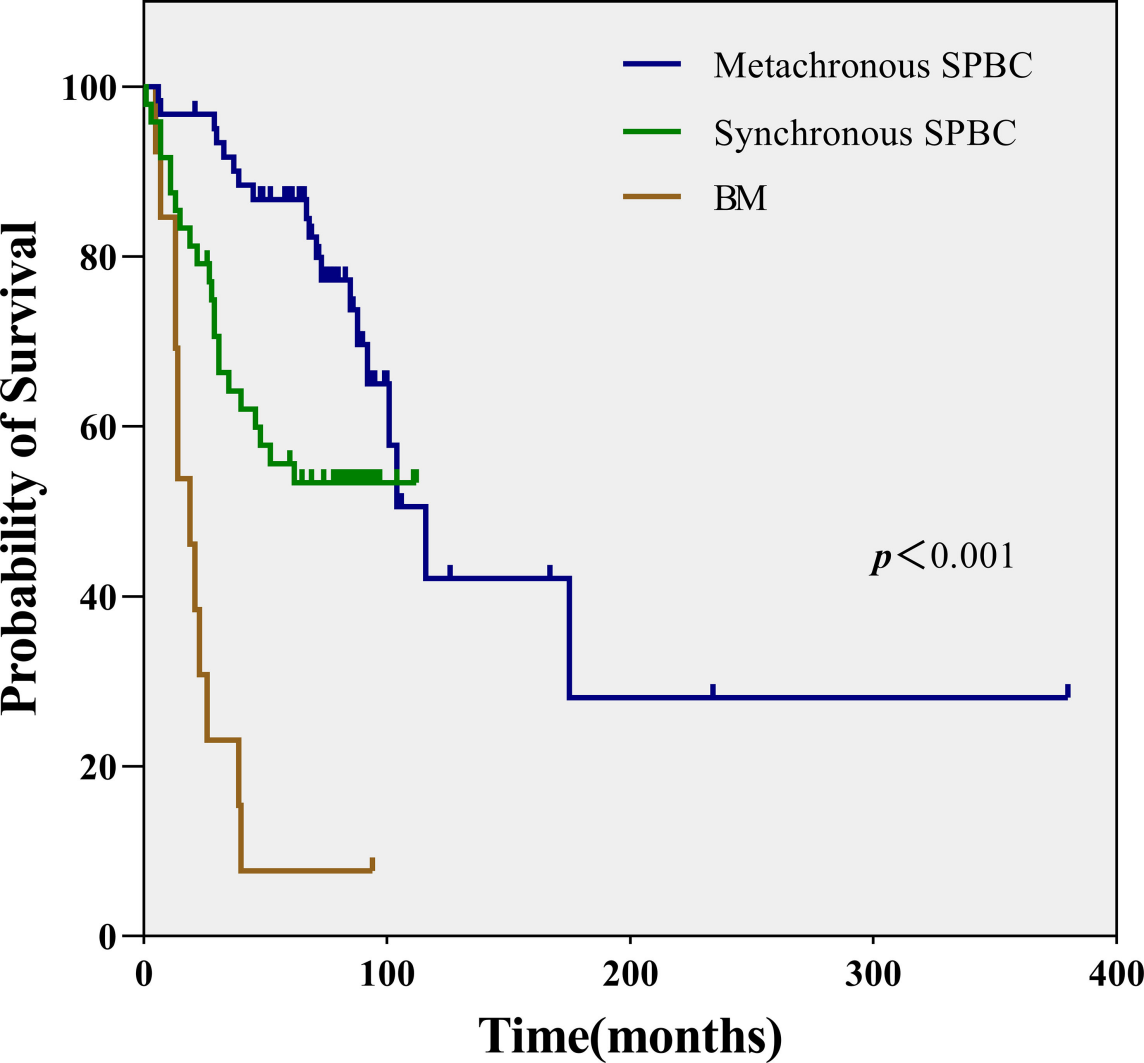
Survival comparison of metachronous group, synchronous group and breast metastases group.

**Figure 3.1:**
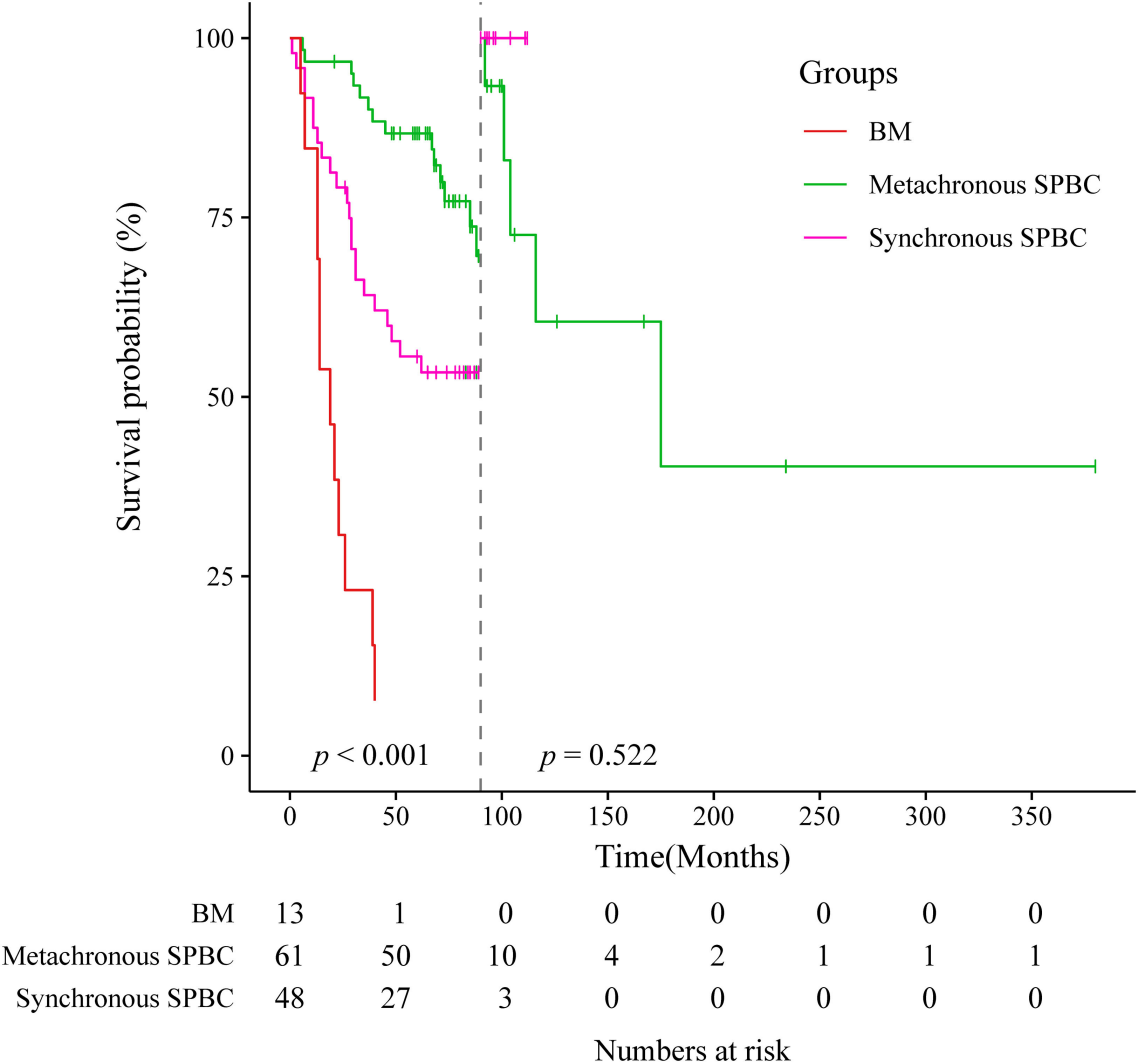
Landmark analysis at the 90-month landmark point of metachronous group, synchronous group and breast metastases group.

Of the 123 patients with SPBC, 109 were followed up, including 48 in the synchronous group and 61 in the metachronous group. The median overall survival time was 71 months (range, 1-380 months). We also followed up 13 patients with BM. Survival data of these patients were collected and analyzed. As shown in **Figures 3**, **3.1**, patients with metachronous SPBC had the longest median survival time (MST) of 72 months, followed by patients with synchronous SPBC, with an MST of 63.5 months. Within 90 months, the prognosis of metachronous SPBC patients was better than that of synchronous SPBC patients (*p*=0.014); however, after 90 months, there was no significant difference in overall survival between the two groups (*p*=0.522).BM patients showed the worst prognosis, with a significantly shorter MST of 19 months (range, 5-94 months) than patients with synchronous SPBC and metachronous SPBC (MST: 19, 63.5, 72 months, *p*<0.001).

Univariate survival analysis showed that ER/PR -negative status, an interval of less than 6 months between the onset of two tumors, a late stage of first primary malignancy, and an age of diagnosis of first primary malignancy greater than 60 years predicted a worse prognosis for patients with SPBC. Multivariate analysis showed that OS improved in patients with ER/PR -positivity and at an early stage when diagnosed with extramammary malignancy (**Table 4**).

**Table 4:** Univariate survival analysis and multivariable analysis of patients with second primary breast carcinoma.

Overall survival		Univariate analysis	Multivariable analysis		
		*P* value	HR	95% CI	*P* value
Gender	Male	0.752			
	Female				
Age	<35	0.774			
	≥35				
Size of breast mass (cm)	<2	0.108			
	2-5				
	>5				
Number of breast tumors	Solitary	0.313			
	Multiple(Unilateral or bilateral breast)				
Ipsilateral axillary lymph node enlargement	Yes	0.546			
	No				
ER/PR status	Positive	0.001	0.427	0.218-0.836	0.013
	Negative				
HER-2 status	Positive	0.450			
	Negative				
Smoking history	Yes	0.612			
	No				
Family history of cancer	Yes	0.978			
	No				
History of radiotherapy or chemotherapy	Yes	0.340			
	No				
Symptoms	Yes	0.421			
	No				
Distant metastasis	Yes	0.344			
	No				
Interval time	Synchronous	0.019	1.678	0.873-3.228	0.121
	Metachronous				
Stage of first malignancy	I/II	<0.001	0.412	0.207-0.822	0.012
	III/IV				
Age at diagnosis of first malignancies	<60	0.006	0.559	0.285-1.093	0.089
	≥60				

### Differential diagnosis of SPBC and BM

A comparison of the clinical characteristics of SPBC and BM is shown in **Table 1**. Patients with SPBC were older than those with BM, with median ages of 55 and 44 years, respectively (*p*=0.018). The positive rate of ER/PR in SPBC patients was significantly higher than that in patients with BM (76.42% *vs.* 5.88%, *p* < 0.001). More patients with BM chose chemoradiotherapy than those with SPBC for the treatment of first primary cancers (76.47% *vs.* 47.15%, *p*=0.023).For the first primary malignant tumor, patients in stage III/IV were a small fraction (28.46%) in SPBC group, whereas they were the majority (88.24%) in BM group (*p* < 0.001). Of the BM patients, 88.24% (15/17) were found to have other organs or lymph node metastasis at the same time as breast metastasis, and only 0.81% (1/123) of SPBC patients had distant metastasis (*p* < 0.001). In terms of the age at diagnosis of first primary malignancy, the mean age of the patients in the SPBC group was older than that of patients in the BM group (53 years *vs.* 48.5 years, *p*<0.001).

When it comes to imaging differences between SPBC and BM patients, the number of breast tumors and the presence of microcalcifications are the main points of difference. As shown in **Table 5**, both SPBC and BM patients were more likely to show a solitary breast tumor. The difference is that patients with SPBC has a higher proportion of solitary breast tumors than BM patients (90.24% *vs.* 64.71%, *p*=0.01). Mammography was performed in 100 patients with SPBC and seven patients with BM. Microcalcifications were found in 75% (75/100) of the patients with SPBC, whereas in the BM group, only one patient with ovarian cancer reported the presence of microcalcifications (14.29%) (*p*=0.002). All 17 patients with BM underwent imaging studies, and four patients were diagnosed with breast metastasis; however, up to 70.59% were diagnosed with primary breast cancer. It was challenging to distinguish between primary breast cancer and metastatic breast cancer on imaging.

**Table 5:** Radiological characteristics of patients with second primary breast carcinoma and with those of breast metastases.

		Second primary breast carcinoma (n=123), n (%)	Breast metastases (n=17), n (%)	*P* value
Radiological diagnosis	Primary	107 (86.99%)	12 (70.59%)	<0.001
	Metastasis	0 (0.00%)	4 (23.53%)	
	Benign	8 (6.50%)	1 (5.88%)	
	Unsure	8 (6.50%)	0 (0.00%)	
Location of breast tumor	Left breast	56 (45.53%)	9 (52.94%)	0.060
	Right breast	61 (49.59%)	5 (29.41%)	
	Bilateral breasts	6 (4.88%)	3 (17.65%)	
Quadrant of breast tumor^a^	Inner quadrant	22 (18.80%)	2 (14.29%)	0.554
	Outer Quadrant	79 (67.52%)	12 (85.71%)	
	Areola	14 (11.97%)	0 (0.00%)	
	Diffuse lesion	2 (1.71%)	0 (0.00%)	
Number of breast tumors	Solitary	111 (90.24%)	11 (64.71%)	0.010
	Multiple (Unilateral or bilateral breast)	12 (9.76%)	6 (35.29%)	
Ipsilateral axillary lymph node enlargement	Yes	44 (35.77%)	7 (41.18%)	0.664
	No	79 (64.23%)	10 (58.82%)	
Microcalcification^b^	Yes	75 (75.00%)	1 (14.29%)	0.002
	No	25 (25.00%)	6 (85.71%)	

a Bilateral breast lesions were excluded;

b Only patients who had undergone mammography were included.

## Discussion

Cancer morbidity is on the rise, and it is estimated that by 2030, more than 21 million people will be diagnosed with malignancies, with morbidity increasing by 1.7% annually (12, 16, 17). Breast cancer is the most common malignancy among women in the United States (18).The number of cancer survivors is increasing owing to advances in diagnosis and treatment. By 2040, an estimated 26.1 million people in the United States will be cancer survivors (19).However, cancer survivors, who make up 3.5% of the U.S. population, make up 10% of newly diagnosed cancer patients, resulting in a growing number of MPMTs (9).A substantial number of patients newly diagnosed with breast carcinoma had different types of malignancies in the past (20).The development of MPMTs may be caused by a variety of genetic, therapeutic, extrinsic, and intrinsic factors (21, 22). Genome-wide association studies have identified 72 loci associated with breast cancer susceptibility, 17 of which are associated with MPMTs (23). Studies have shown that survivors of breast malignancy have a 25% increased risk of developing extramammary primary malignancies (10, 11). The association between malignant breast tumors and non-breast primary cancers has also been well documented (24–31). However, little attention has been paid to primary breast cancers secondary to non-breast primary malignancies.

Some studies have confirmed the pathogenesis of primary breast cancer secondary to primary extramammary malignancies. In terms of the type of first primary extramammary malignancy in patients with SPBC, we observed that thyroid cancer was the most common, accounting for 30.08% (37/123). In fact, several studies have reported elevated SPBC morbidity in thyroid cancer survivors (32, 33). The rising morbidity of thyroid cancer and the generally long survival time of thyroid cancer patients have led to a growing number of thyroid cancer survivors, whose long survival time also gives them enough time to develop a second primary malignancy, such as breast cancer. One of the molecular mechanisms underlying the increased risk of SPBC in thyroid cancer survivors could be the high ER levels in thyroid cancer patients, which are generally higher than those in the general population with sex steroid receptors in the human thyroid tissue (34, 35).In our study, there were 28 cases of gynecological tumors, which were the second most common extramammary malignancy after thyroid cancer, accounting for 22.76% of SPBC patients. It has been proven that gene mutation plays an important role in the occurrence of gynecologic tumors and breast cancer. Patients with *BRCA* or *TP53* mutations have a significantly higher lifetime risk of both ovarian and breast cancers than the general population (36, 37), which is one of the reasons why patients with gynecologic neoplasms are more likely to develop SPBC. Unfortunately, due to the economic conditions of the patients, most of them were not tested for gene mutations, which made it impossible to obtain the mutation data of *BRCA* or *TP53* in these patients. Furthermore, in addition to the same causative genes, another reason for the elevated risk of SPBC in gynecologic cancer survivors may be the higher survival rate of patients with gynecologic tumors, which has also been shown in previous studies conducted in Taiwan, China (38, 39).In our study, thyroid and gynecologic cancers accounted for more than half of the primary malignant tumor types in SPBC patients; therefore, understanding the potential association of these two tumor types with breast cancer could provide some clinical experience and evidence to us. Notably, a strong association between melanoma and breast cancer has been frequently reported. Both melanoma and breast cancer cells exhibit ERs, and hormonal factors in melanoma survivors may contribute to the development of SPBC (40–42). However, no patients with melanoma were observed in our study, possibly because of the high incidence of cutaneous melanoma in white women but less often in Asian populations (43). A previous study in Korea found an increased risk of SPBC in patients with primary bladder cancer (44), and it was hypothesized that germline mutations in fumarate hydratase might be associated with this phenomenon (45).Bluhm et al. found that the risk of SPBC was highest 5-9 years after NHL diagnosis (46), while another study showed that SPBC was significantly increased 10 years after NHL diagnosis (43).This may be due to the long-term effects of chemoradiotherapy in lymphoma patients.

An increased risk of breast cancer has been observed in many types of cancer within the first five years following diagnosis. Our study found that more than half of the patients (55.88%) in the metachronous group had two different types of primary malignancies within 5 years. Similar to our findings, Sandi et al., studying 10822 patients diagnosed between 2005 and 2015, found that 46.3% of the patients developed breast cancer within 5 years of the incident previous cancer diagnosis (47). Although the risk of breast cancer is high within 5 years of the occurrence of extramammary tumors, we found that the risk decreases over time.

Both primary and metastatic breast cancers should be considered when a cancer patient has a breast mass. Metastatic breast cancer is rare because the breast is rich in fibrous tissue and has a relatively poor blood supply. Williams et al. (48) reported data from 169 patients with breast metastases from solid tumors outside the breast, in which the most common primary tumor was malignant melanoma. Lee et al. (49) reported data from 33 patients with breast metastases from extramammary malignancies, in which the most common primary tumor was gastric cancer. In our study, the most common primary tumors in BM patients were lung cancer (35.29%), followed by ovarian cancer (11.76%), lymphoma (11.76%) and rectal cancer (11.76%), with rare primary tumors including hepatic carcinoma, gastric carcinoma, renal carcinoma, mucoepidermoid carcinoma, and malignant schwannoma in one case each. This result may reflect the different occurrences and treatments of malignant tumors in different races, regions, and hospitals. Due to the rarity of metastatic breast cancer, there are insufficient data to differentiate primary breast cancer from metastatic breast cancer. Both primary and metastatic breast cancers are characterized by painless solitary breast nodules, which are usually located in the outer quadrant of the breast because of the relatively abundant blood supply and glands in the outer quadrant. Metastatic breast cancer has a rapid nodule growth rate, and skin changes and nipple discharge are very rare (50–52). This finding is consistent with the results of the present study. McCrea et al. (53) suggested that microcalcifications are almost absent in patients with breast metastases, except in those whose primary tumor is ovarian cancer, where microcalcifications are mostly present. In this study, we found one case of metastatic breast cancer with microcalcification, the primary tumor of which was ovarian cancer. Therefore, it is inaccurate to distinguish breast nodules as primary or metastatic based on the presence or absence of microcalcifications. In this study, we found that patients with metastatic breast cancer were younger than those with SPBC, which may be due to the richer blood supply to the breast in younger patients. ER/PR-positive rates in patients with BM are usually low because these receptors are usually present in the breast tissue. Breast metastasis is mostly a part of systemic metastasis. In this study, most patients (88.24%) presented with additional systemic metastases when breast metastases were found. Patients with breast metastasis usually lose the chance of surgery and need systemic treatment for the primary tumor, while patients with SPBC are usually staged earlier and treated with comprehensive treatment based on surgery. Due to the different treatment methods, it is particularly important to make a good differential diagnosis between the two.

A previous study (43) has shown that the proportion of female patients with SPBC who survived for more than 5 years was approximately 43.82%, while in our study, the proportion was 66.06% (median OS:71 months), which may be due to the improvement of people’s health awareness and more targeted follow-up after the diagnosis of the first tumor. In addition, it may also reflect differences in the predilection type and malignancy of the first tumor in patients of different countries and races. Williams et al. (48) reported a median survival of 10 months for 169 cases of breast metastases from extramammary solid tumors; in our study, the median survival from the time BM was diagnosed was 19 months. Breast metastasis is rare, and we hope that more relevant studies will emerge in the future to provide more references.

## Conclusions

In conclusion, SPBC is not uncommon, and survivors of extramammary malignancies should be followed-up. Therefore, it is important to make a differential diagnosis between SPBC and BM. The ER/PR status and the first primary malignancy may affect the prognosis of patients with SPBC. Identifying the characteristics of different coexisting primary cancers may increase the clinical vigilance of physicians and warrant new screening procedures to detect certain second primary malignancies at an earlier stage in patients with malignant tumors. However, there are still some limitations in this study. In this study, a number of factors that may affect the prognosis of SPBC patients were obtained through retrospective study, but the conclusions were not further verified. We look forward to developing a risk early warning system for cancer patients in the future by combining with engineers and artificial intelligence.

## Data availability statement

The raw data supporting the conclusions of this article will be made available by the authors, without undue reservation.

## Ethics statement

The studies involving human participants were reviewed and approved by Medical Ethics Committee of Tianjin Medical University Cancer Institute&Hospital. The patients/participants provided their written informed consent to participate in this study.

## Author contributions

YJ performed the analysis and wrote the manuscript. YJ modified the article. XW and BS designed and supervised the research. XW and BS contributed equally to this research. All authors examined and accredited the final manuscript. All authors contributed to the article and approved the submitted version.
